# Using Tweets to Understand How COVID-19–Related Health Beliefs Are Affected in the Age of Social Media: Twitter Data Analysis Study

**DOI:** 10.2196/26302

**Published:** 2021-02-22

**Authors:** Hanyin Wang, Yikuan Li, Meghan Hutch, Andrew Naidech, Yuan Luo

**Affiliations:** 1 Department of Preventive Medicine Northwestern University Chicago, IL United States; 2 Department of Neurology Northwestern Universtity Chicago, IL United States

**Keywords:** COVID-19, social media, health belief, Twitter, infodemic, infodemiology, machine learning, natural language processing

## Abstract

**Background:**

The emergence of SARS-CoV-2 (ie, COVID-19) has given rise to a global pandemic affecting 215 countries and over 40 million people as of October 2020. Meanwhile, we are also experiencing an infodemic induced by the overabundance of information, some accurate and some inaccurate, spreading rapidly across social media platforms. Social media has arguably shifted the information acquisition and dissemination of a considerably large population of internet users toward higher interactivities.

**Objective:**

This study aimed to investigate COVID-19-related health beliefs on one of the mainstream social media platforms, Twitter, as well as potential impacting factors associated with fluctuations in health beliefs on social media.

**Methods:**

We used COVID-19-related posts from the mainstream social media platform Twitter to monitor health beliefs. A total of 92,687,660 tweets corresponding to 8,967,986 unique users from January 6 to June 21, 2020, were retrieved. To quantify health beliefs, we employed the health belief model (HBM) with four core constructs: perceived susceptibility, perceived severity, perceived benefits, and perceived barriers. We utilized natural language processing and machine learning techniques to automate the process of judging the conformity of each tweet with each of the four HBM constructs. A total of 5000 tweets were manually annotated for training the machine learning architectures.

**Results:**

The machine learning classifiers yielded areas under the receiver operating characteristic curves over 0.86 for the classification of all four HBM constructs. Our analyses revealed a basic reproduction number *R*_0_ of 7.62 for trends in the number of Twitter users posting health belief–related content over the study period. The fluctuations in the number of health belief–related tweets could reflect dynamics in case and death statistics, systematic interventions, and public events. Specifically, we observed that scientific events, such as scientific publications, and nonscientific events, such as politicians’ speeches, were comparable in their ability to influence health belief trends on social media through a Kruskal-Wallis test (*P*=.78 and *P*=.92 for perceived benefits and perceived barriers, respectively).

**Conclusions:**

As an analogy of the classic epidemiology model where an infection is considered to be spreading in a population with an *R*_0_ greater than 1, we found that the number of users tweeting about COVID-19 health beliefs was amplifying in an epidemic manner and could partially intensify the infodemic. It is “unhealthy” that both scientific and nonscientific events constitute no disparity in impacting the health belief trends on Twitter, since nonscientific events, such as politicians’ speeches, might not be endorsed by substantial evidence and could sometimes be misleading.

## Introduction

Beginning in December 2019, the outbreak of SARS-CoV-2 rapidly evolved into a global pandemic [1-3]. As of the writing of this paper, over 40 million cases and 1 million deaths from 215 countries or regions have been confirmed [4]. However, spreading faster than the virus is information. Sylvie Briand, Director of Infectious Hazards Management at the World Health Organization (WHO)’s Health Emergencies Programme, pointed out “We know that every outbreak will be accompanied by a kind of tsunami of information, but also within this information you always have misinformation, rumors, etc.” [5]. The WHO used the term *infodemic* to describe the overabundance of information and misinformation occurring during the COVID-19 pandemic. Though the term infodemic was first coined in 2002 [6], the concerns over infodemics have recently become dramatic with the amplification effect from social media. The WHO held the first Infodemiology Conference in June 2020 as the phenomenon had escalated to a level that required a coordinated response [7]. Even though we cannot avoid an infodemic, we can still manage it. Previous studies and commentaries proposed several perspectives to detect and fight the COVID-19 infodemic [5,8-10]. However, one of the critical points absent from these studies is an investigation of health beliefs. Understanding how the general public’s health beliefs are expressed and altered can facilitate our management of both the pandemic and infodemic. In conjunction, it is also essential to evaluate any concurrent or ongoing interventions.

The health belief model (HBM) quantifies health beliefs [11-13]. The HBM was developed to investigate people’s beliefs about health problems. It consists of the following four core constructs that can be tailored for given hypotheses: (1) perceived susceptibility, (2) perceived severity, (3) perceived benefits, and (4) perceived barriers. The HBM has been widely used to investigate individual opinions toward diseases and interventional approaches, such as HIV risk behaviors [14], human papillomavirus (HPV) vaccines [15], and the gender difference in food choices [16]. In those cases, the HBM was employed to evaluate people’s beliefs toward the given health problem and their perceived benefits or barriers of action, for which each of the core constructs of the HBM is assessed based on the corresponding definitions. During pandemics, researchers have employed the HBM to investigate the health beliefs toward public interventional policies, such as stay-at-home orders [17]; to analyze public health communication on Instagram during the Zika outbreak [18]; to examine public perceptions of physical distancing [19]; and to guide community pharmacists in their communication with patients [20]. However, because these are survey-based or merely commentary studies, results are limited to the analyzed population and, therefore, may be biased. In this study, we expanded and diversified our study population by using crowdsourcing data from one of the mainstream social media platforms, Twitter, in order to investigate the health beliefs of the general public toward COVID-19 and its potential treatments.

In addition to quantifying health beliefs, we aimed to identify factors influencing fluctuations in public opinions. For instance, the pandemic dynamics (ie, the number of cases and deaths due to COVID-19) constitute one of the leading factors influencing attitudes toward the pandemic. Additionally, interventional government policies may also impact the opinions of the general public. Furthermore, it is reasonable to believe that health belief–related posts can also be self-regulated as a consequence of their nature to induce or soothe panic for readers. Potential treatments trigger massive discussions as well, such as the debate over the appropriate use of the antimalarial drug hydroxychloroquine (HCQ) or chloroquine (CQ), advocated for by the then American President as a “game changer,” which was then subsequently discarded. Public attitudes regarding potential treatments may be altered by public events, such as the news or politicians’ speeches. Furthermore, rapidly emerging scientific publications can also influence the point of view of the general public. In this paper, we aim to identify factors that impact health beliefs on social media, which may serve as a probe for identifying better strategies to manage both the pandemic and the infodemic.

Contributions of this study include the following:

1. An evaluation of utilizing a mainstream social media platform, Twitter, to facilitate a comprehensive understanding of health beliefs toward COVID-19 and potential treatments.
2. A publicly available data set annotated by multiple professionals for studying the health beliefs related to COVID-19 and potential treatments.
3. Identification and comparison of factors that influence health beliefs toward COVID-19 and potential treatments, HCQ or CQ in particular.
4. An extendable framework for monitoring the general public’s health beliefs during a pandemic and infodemic, which could be feasibly transferred to facilitate the management of future infodemic outbreaks, such as when COVID-19 vaccines become available to the public.

## Methods

**Figure 1:**
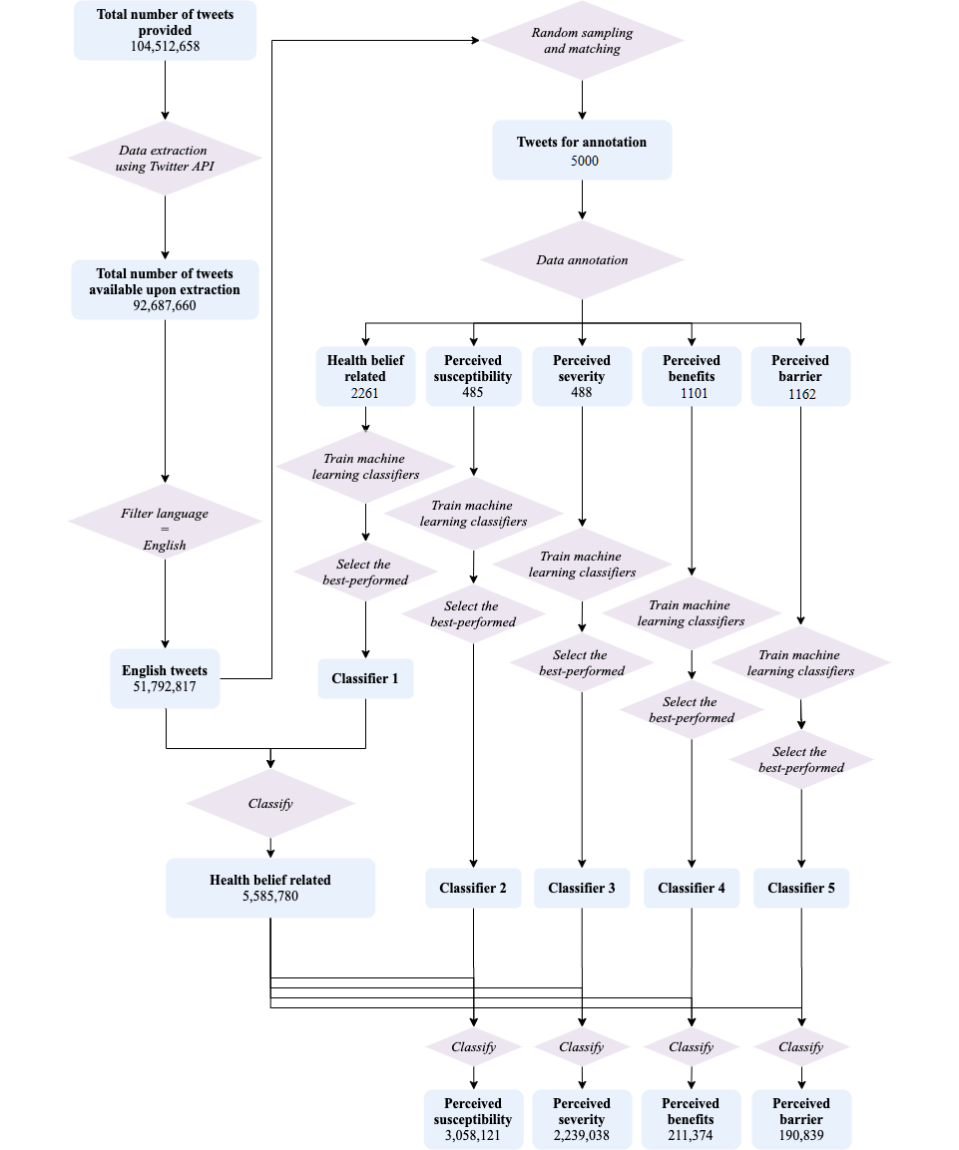
The workflow of data selection, data annotation, and machine learning classifier training and implementation. API: application programming interface.

The entire workflow of data extraction, filtering, and classification is illustrated in Figure 1.

### Data

We used version 15.0 of the COVID-19 Twitter chatter data set constructed by the Panacea Lab [21], which collected all COVID-19-related tweets between January 6 and June 21, 2020. The provided data set only contains the identifiers of corresponding tweets, so we used the application programming interface (API) provided by Twitter to extract the full content of each tweet. The Social Media Mining Toolkit provided by the Panacea Lab was used to hydrate the data set [22]. There is no limitation regarding the days prior to the extraction. A language filter (ie, “lang” attribute in the tweet object = “en”) was then applied to identify tweets written in the English language.

### Data Annotation for Constructing the Health Belief Model

We employed the HBM to quantify health beliefs. As mentioned above, it consists of the following four core constructs that can be tailored for given hypotheses: (1) perceived susceptibility, (2) perceived severity, (3) perceived benefits, and (4) perceived barriers. The HBM was developed to investigate people’s beliefs about health problems and has been widely used to investigate individual opinions toward diseases and interventional approaches, such as HIV risk behaviors [14], HPV vaccines [15], and the gender difference in food choices [16]. Specifically, for perceived benefits and barriers, we focused on HCQ or CQ, the antimalarial drug advocated for by the then American President as a “game changer,” which was then subsequently discarded. Tweets were labeled as positive or negative for being related to HBM, meaning they could be mapped to at least one of the four aforementioned constructs. Thus, each tweet could potentially have up to five labels. The annotation process was performed by three senior PhD students in biomedical informatics (HW, YLi, and MH). All annotators classified the first 500 tweets individually, then reconciled different opinions and built final annotation rules. The definitions for each construct of the HBM are described in Table 1 with example tweets. Based on the rule, HW and YLi annotated the rest of 5000 tweets independently and evaluated the agreement using the Cohen κ score [23]. Finally, MH resolved the divergent annotations between HW and YLi with further consideration. We made the data set with the 5000 annotated tweets available for researchers [24]. To protect the privacy of Twitter users and per Twitter’s policy, we did not include any tweet content in the data set. Instead, each tweet’s unique identifier (ie, tweet ID) was provided.

**Table 1:** Health belief model (HBM) constructs, definitions, and example tweets.

Construct	Definitions of the construct	Example tweet
Perceived susceptibility	The assessment of the risk of getting COVID-19 infection	“Across the UK, 194,990 people had tested positive for coronavirus as of 9am on Tuesday, up from 190,584 at the same point on Monday. Find out how many cases there are in your area.”
Perceived severity	The assessment of whether COVID-19 is a sufficient health concern	“US Recorded 1,297 Coronavirus Deaths in Past 24 Hours.”
Perceived benefits	The benefits of HCQ^a^ and CQ^b^ in prevention or treatment of COVID-19 Positive statements or reports about HCQ and CQ	“Dr. Zelenko In NY has now treated 699 Coronavirus patients with 100% success using Hydroxychloroquine.”
Perceived barriers	The side effects of HCQ and CQ The unaffordable cost of HCQ and CQ The inaccessibility of HCQ and CQ Negative statements or reports about HCQ and CQ	“Family of New York woman blames hydroxychloroquine combo for fatal heart attack.”
HBM related	Can be mapped to at least one of the four constructs above	Any of the above tweets are examples for this construct

^a^HCQ: hydroxychloroquine.

^b^CQ: chloroquine.

### Machine Learning Classifiers

We trained machine learning classifiers on the annotated data, evaluated the performance, and automatically classified the 50 million tweets. The entire annotated data set was split into a training data set and a testing data set with a ratio of 8:2. Feature selection was applied by ignoring terms with a document frequency of less than 0.01 or greater than 0.99. Terms with only letters were considered, but terms were ignored when there were numbers or special characters. Before vectorization, we removed all the URLs; unified all the contractions, punctuation marks, and white spaces; and converted all terms to lowercase in the corpus. The free-text tweets were vectorized using both bag-of-words and term frequency–inverse document frequency (tf-idf) algorithms. A list of English stop words provided by the natural language toolkit [25] was used to rule out unrelated words. Next, 5-fold cross-validation was performed to select the best-suited classifier for the task. The machine learning classifiers that we experimented on included Ridge classifier; perceptron; passive-aggressive classifier; k-nearest neighbors classifier; random forest; support vector machine with linear kernel and l1 or l2 penalty; support vector machine with radial basis function, polynomial, or sigmoid kernel; stochastic gradient descent classifier with l1 or l2 or elastic net penalty; multinomial naïve Bayesian classifier; Bernoulli naïve Bayesian classifier; and logistic regression. Performance across classifiers was evaluated using the area under the receiver operating characteristic (AUROC) curve, and the classifier that yielded the highest AUROC was chosen (see Multimedia Appendix 1, Tables S1-S5). In total, five classifiers were built to construct the final HBM. First, a classifier was trained to classify whether a tweet was HBM related or not. Then, we collected all the tweets that were identified as HBM related for the following task. Lastly, we built four classifiers to label each core construct of the HBM separately.

The entire pipeline was built with Python 3.6.8 (Python Software Foundation). The bag-of-words algorithm, machine learning classifiers, and model evaluations were implemented with the scikit-learn, version 0.22.1, package [26].

### The Overall Trend of Health Beliefs in Tweets

To quantify whether the information spread constituted an infodemic, we applied one of the classic measurements in epidemiological models: the basic reproduction number, *R*_0_. We employed the susceptible-infectious-recovered (SIR) model [27], for which the detailed calculation can be found in the Multimedia Appendix 1. In our case of an infodemic, we considered the users who tweeted about COVID-19 as the susceptible population; among this population, *being infected* meant a user tweeted about health beliefs defined in our HBM scope, and *recovering* then indicated that a user stopped tweeting about health beliefs. Thus, *contact with infected individuals* could be considered as reading health belief–related tweets posted by other users.

### The Trend of Health Belief Toward the Disease

**Figure 2:**
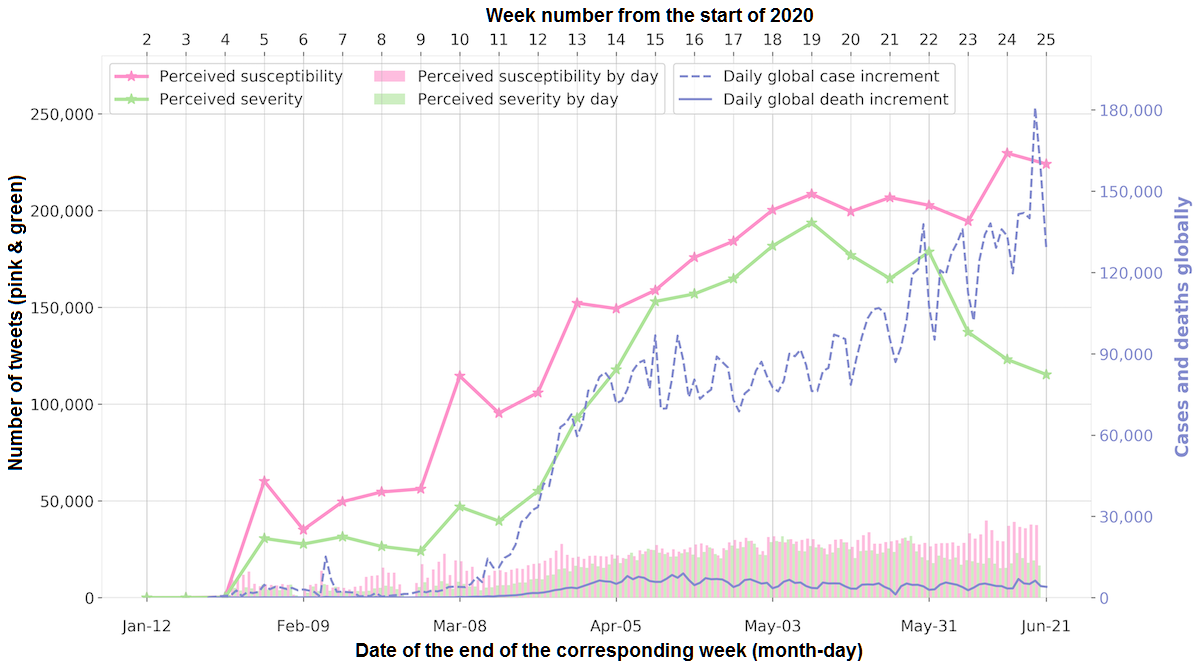
Dynamics of perceived susceptibility and severity with COVID-19 case and death trends. The pink and green lines with "star" marks reflect the weekly cumulative number of tweets for perceived susceptibility and severity, while the pink and green bars on the x-axis indicate the daily number of tweets related to perceived susceptibility and severity. The global case and death dynamics have been available since January 22, 2020.

There are two core constructs in the HBM that focus specifically on the disease of interest: perceived susceptibility and perceived severity. We visualized these two constructs together with the dynamics of the pandemic in Figure 2. We observed a similar pattern in COVID-19 case dynamics and the number of tweets regarding perceived susceptibility, as well as in the dynamics of COVID-19 deaths and the number of tweets indicating perceived severity. For the first pair, we observed an earlier increase in the number of perceived susceptibility tweets prior to a surge in COVID-19 cases, while for the second pair, there was a delay in the increase of COVID-19 deaths compared with the number of perceived severity tweets. To investigate how many days the trend dynamics of health belief discussions followed or postponed the actual case or death increases, we calculated the Spearman correlation coefficient under various time lags (ie, for a 1-day lag, the correlation between the number of tweets and the COVID-19 situation was calculated by moving the COVID-19 trend 1 day forward). Moreover, we conducted a change point analysis using the dynamic programming algorithm [28] to detect the significant turning point of the trends.

### The Effect of Interventions

To evaluate the impact of interventions on the infodemic and the pandemic, we further investigated the lockdown in the United States. Because we were analyzing only tweets written in the English language, and there were systematic official lockdowns issued in the United States, we chose to study the effect of US-based interventions on health beliefs. The location information is not available for each tweet. We analyzed 136,641 tweets where the *place* was available—a variable in the tweet object that, when present, indicates that the tweet is associated with a place—and subsequently identified 54,164 tweets corresponding to the United States. We investigated the effect of interventions by visualizing the trends along with the timeline of lockdowns in the United States.

### News in the Top Topics

To understand major topics in the tweets related to health beliefs, we extracted the top 10 phrases from the tweets each week. We considered unigrams and bigrams in this case. The frequency of each phrase was not only calculated as the count; instead, we used the tf-idf score to find the highlighted topics of each week. A higher tf-idf score is obtained if a given word or phrase frequently appears in one document but only appears in a small number of documents.

### The Influence of Scientific and Nonscientific Events

To evaluate the difference between the impact of scientific and nonscientific events on health beliefs, we conducted a Kruskal-Wallis test. The Kruskal-Wallis test was chosen since there was no reasonable assumed distribution for the influence of the two types of events, and the two groups being compared had different sample sizes. We collected events associated with HCQ and CQ on the internet during the study period with no exclusion criteria. All the events were classified as scientific events if they were based on scientific evidence or endorsed by authorities, while all remaining events were treated as nonscientific events. To quantify the influences, for each event, we calculated the sum of the number of tweets that expressed perceived benefits or perceived barriers regarding HCQ and CQ on the day of the event and the day after.

## Results

### Data and Machine Learning Classifiers

The data set contained identifiers for 104,512,658 unique tweets, of which 92,687,660 were still available upon extraction. After applying the language filter, our final set for analysis consisted of 51,792,817 English tweets. The Cohen κ score for interrater reliability of data annotation was 0.94 for identifying whether a given tweet was HBM related and was around 0.9 for the annotation of all four individual HBM constructs (see Table 2). Random forest was found to be the best-performing classifier for the HBM-related classification and three of the HBM constructs (ie, perceived susceptibility, perceived benefits, and perceived barriers), while the passive-aggressive classifier was found to be the most suitable choice for classifying whether a tweet indicated perceived severity. The AUROC curves for HBM-related and the two disease-related constructs were all above 0.9, while AUROC curves for the two treatment-related constructs were around 0.86 (see Table 2).

After classification, 5,585,780 tweets were HBM related, among which 3,058,121 (54.75%) tweets expressed perceived susceptibility of COVID-19, 2,239,038 (40.08%) tweets expressed perceived severity of COVID-19, 211,374 (0.04%) tweets expressed perceived benefits of HCQ or CQ, and 190,839 (0.03%) tweets expressed perceived barriers toward HCQ or CQ. To further ensure the validity of the classification, we performed additional spot checks on the final results; examples can be found in Multimedia Appendix 1.

**Table 2:** Performance of machine learning classifiers.

Construct	Cohen κ^a^	Classifier^b^	AUROC^c^ curve	Accuracy	Precision^d^	Recall^e^	F1 score^f^
Perceived susceptibility	0.92	Random forest	0.97	0.94	0.92	0.85	0.88
Perceived severity	0.88	Passive-aggressive	0.92	0.90	0.88	0.77	0.81
Perceived benefits	0.92	Random forest	0.87	0.79	0.78	0.78	0.78
Perceived barriers	0.92	Random forest	0.86	0.77	0.77	0.77	0.77
HBM^g^ related	0.94	Random forest	0.90	0.84	0.84	0.84	0.84

^a^The Cohen κ coefficient for interrater reliability of annotation.

^b^The machine learning classifier selected by the best performance.

^c^AUROC: area under the receiver operating characteristic.

^d^Macro-averaged precision.

^e^Macro-averaged recall.

^f^Macro-averaged F1 score.

^g^HBM: health belief model.

### The Overall Trend of Health Beliefs in Tweets

**Figure 3:**
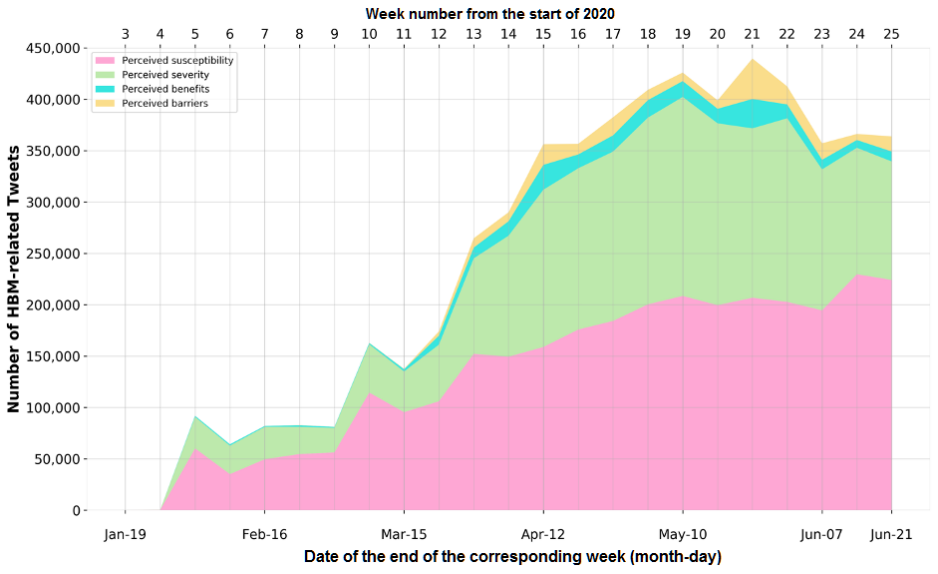
Stacked area chart for the four core constructs of the health belief model (HBM) from January 19 to June 21, 2020.

The visualization of the overall trend of health beliefs is shown in Figure 3*,* with the number of tweets that fell into each core construct of the HBM. Each construct was displayed in a different color chronologically, starting from the third week of 2020, and stacked together to show the total number of HBM-related tweets. A dramatic increase can be observed from January to June, which indicates an increasing number of discussions regarding personal health beliefs. The *R*_0_ was 7.62 for the users who tweeted about health beliefs in our data.

### The Trend of Health Belief Toward the Disease

**Figure 4:**
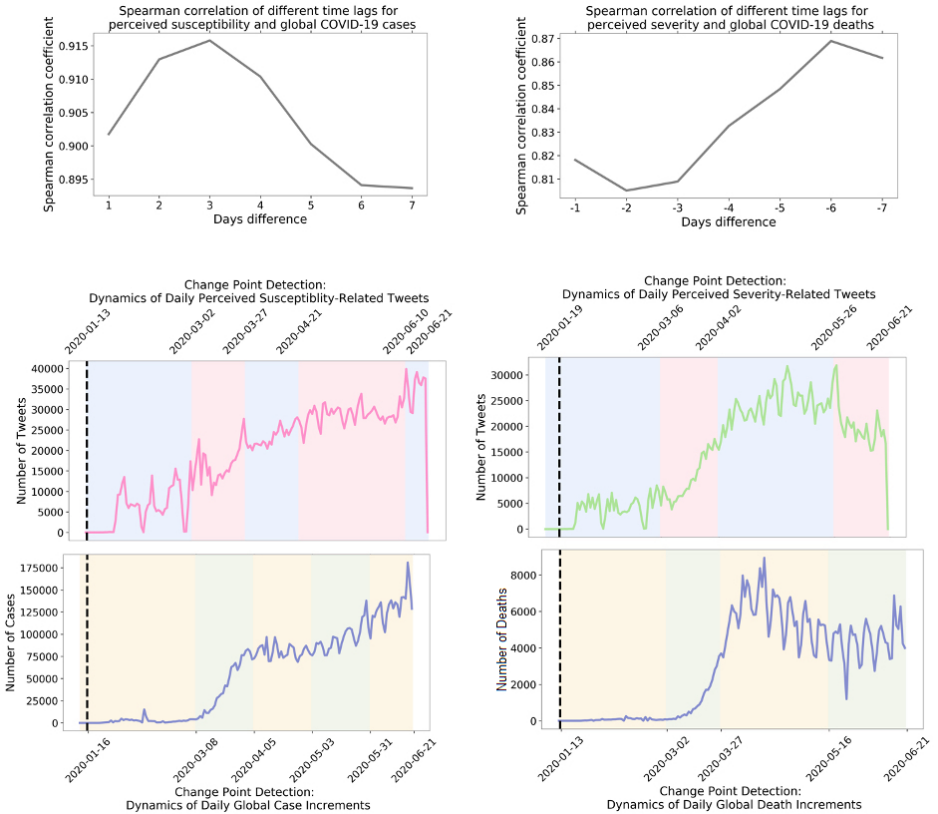
Correlation between perceived susceptibility–related tweets and COVID-19 case dynamics. Each pair of the lower four graphs are staggered according to the time differences that achieve the highest correlation in the top graphs: 3 days and –6 days, respectively. The pink and blue as well as the yellow and green shades depict the change points detected from the change point analysis.

The left panel of Figure 4 displays the strongest correlation (ie, 0.92) between perceived susceptibility–related tweets and the global case increment when imposing a 3-day lag (ie, moving the trend of COVID-19 cases 3 days forward so that the number of perceived susceptibility tweets on January 13, 2020, will be aligned with the number of COVID-19 cases on January 16, 2020). The patterns detected by the change point analysis depicted by color in Figure 4 also show similarities within the pair. For the second pair (ie, perceived severity and COVID-19 death trend in the right-hand panel of Figure 4), the strongest correlation was found at –6 days (ie, 0.87), which indicates that changes in the perceived severity were lagging the actual death dynamics by 6 days (ie, the strongest correlation was found when moving the death trend 6 days backward). The change point analysis unraveled similar patterns between the trends of perceived severity and COVID-19 deaths.

### The Effect of Interventions

**Figure 5:**
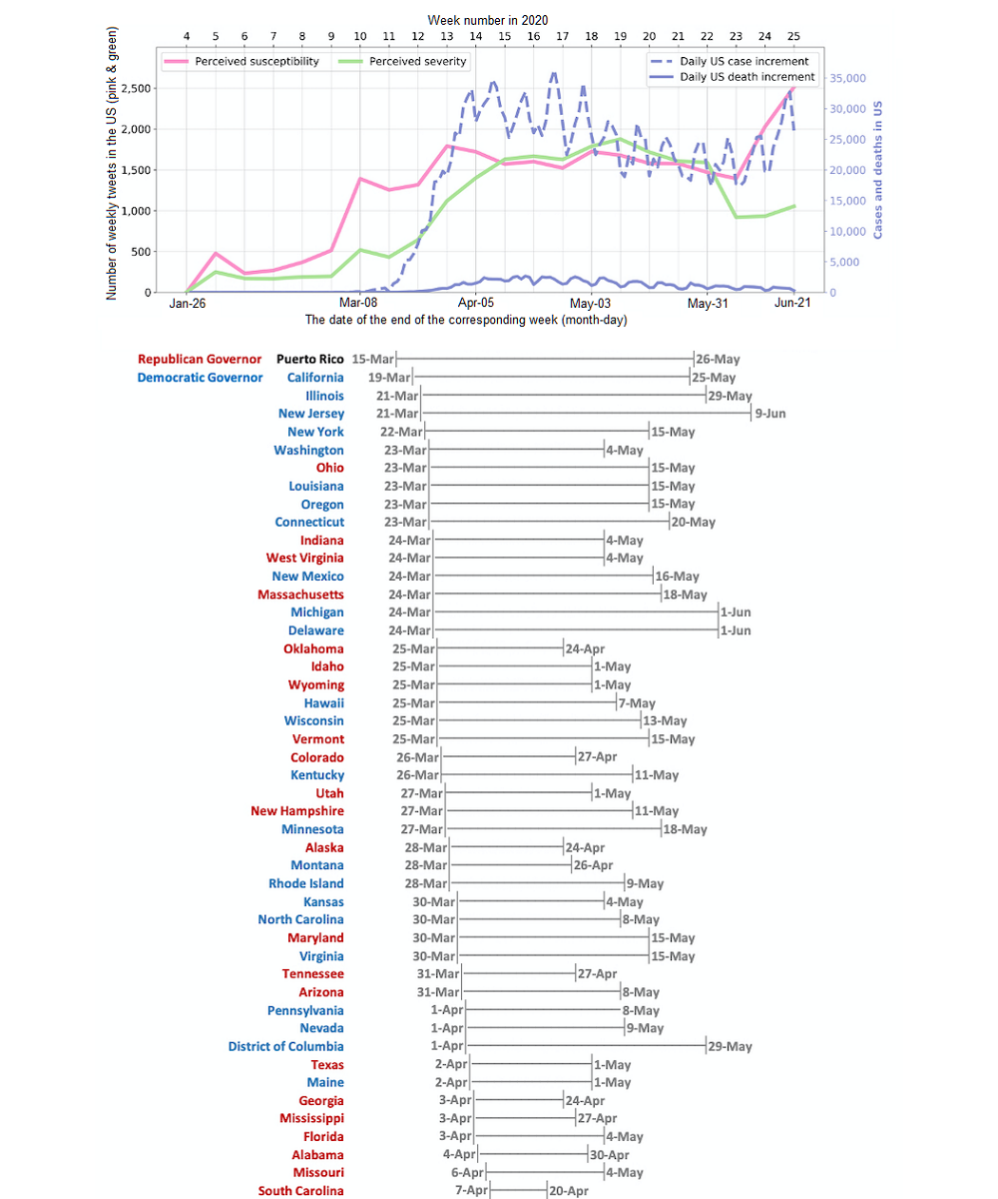
Dynamics of health beliefs related to COVID-19 and the trend of case and death fluctuation in the United States with lockdown status. The lower half of the figure shows the official lockdown circumstances in each US state by each governor. The lines corresponding to each state represent the start and end date of the official lockdowns. The Republican states South Dakota, North Dakota, Iowa, Nebraska, and Arkansas did not announce official lockdowns and are not shown in this figure. The full reference for each state can be found in Multimedia Appendix 1, Table S6.

We visualized the trends of perceived susceptibility and perceived severity along with the daily case and death dynamics in the United States in Figure 5. Meanwhile, lockdown information for each state is also listed by the timeline. Decisions made by Republican or Democratic governors are colored red and blue, respectively. The Republican states South Dakota, North Dakota, Iowa, Nebraska, and Arkansas did not announce official lockdowns and are not included in this figure. The official documents of lockdown and reopen decisions for each state are listed in Multimedia Appendix 1, Table S6.

### News in the Top Topics

**Figure 6:**
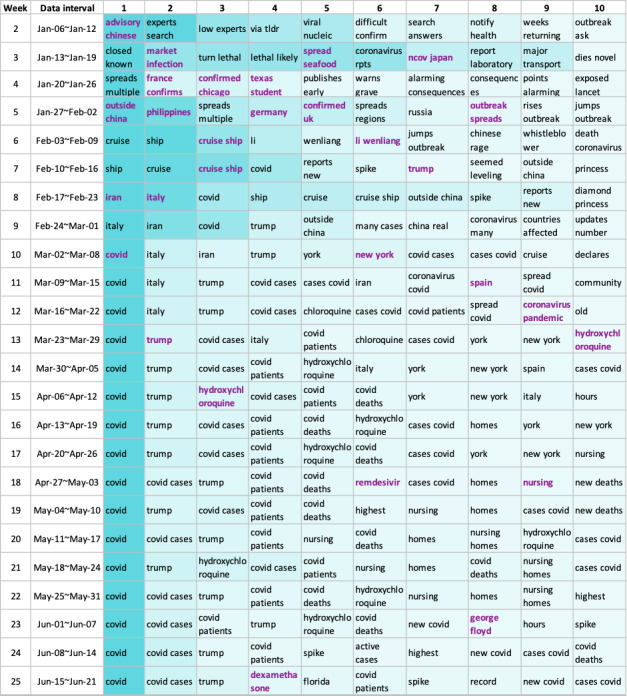
Top 10 topics of each week. Top phrases for each week are organized horizontally in each row. The blue shade in each cell indicates the term frequency–inverse document frequency (tf-idf) score of the phrase; the higher the tf-idf score, the darker the shade. The phrases that were likely associated with news are highlighted in purple.

Top 10 topics, which where all changed to lowercase, according to the tf-idf scores are shown in Figure 6, where a darker shade of the cell indicates a higher tf-idf score. The featured phrases that were closely related to the news during the corresponding time periods are highlighted in purple.

### The Influence of Scientific and Nonscientific Events

**Figure 7:**
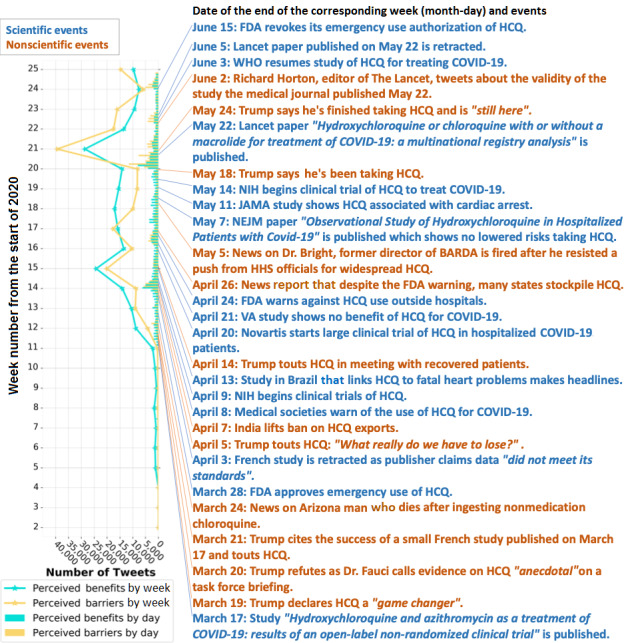
Dynamics of health beliefs related to hydroxychloroquine (HCQ) and chloroquine (CQ) with correlated scientific and nonscientific events. Scientific events are those that have concrete scientific evidence or are endorsed by authorities, while nonscientific events account for the rest. The full reference for each event can be found in Multimedia Appendix 1, Table S7. BARDA: Biomedical Advanced Research and Development Authority; FDA: US Food and Drug Administration; HHS: United States Department of Health and Human Services; JAMA: The Journal of the American Medical Association; NEJM: The New England Journal of Medicine; NIH: National Institutes of Health; VA: United States Department of Veterans Affairs; WHO: World Health Organization.

The list of events that we collected is shown on the right-hand side of Figure 7, while the trends of perceived benefits and barriers are shown on the left-hand side. We observed that both scientific and nonscientific events were associated with fluctuations in health beliefs. The scales of the fluctuations observed varied over time. There were more nonscientific events around the two most massive spikes, but scientific events were majorly distributed along the timeline where many gentle fluctuations could be found. The Kruskal-Wallis test showed no significant difference between the influence of scientific and nonscientific events for both perceived benefits and barriers (*H*=0.078, *P*=.78; and *H*=0.002, *P*=.92, respectively). Full references for each event can be found in Multimedia Appendix 1, Table S7.

## Discussion

### Principal Findings

Through the utilization of natural language processing (NLP) and machine learning, we employed the HBM to identify tweets associated with health beliefs. Through further evaluation of HBM-related tweets, our findings demonstrated that trends in health beliefs were correlated with dynamics in positive case and mortality rates. Additionally, we observed a decline in perceived disease susceptibility during government-issued lockdowns, while perceived severity appeared unaltered. Lastly, our study identified top news events, scientific and nonscientific, that may play a role in altering health beliefs. These findings lay the groundwork to better understand how the general public’s COVID-19-related health beliefs are influenced by case and mortality rates, government policies, current news, and significant events. Through careful study of these observations, we may better implement management strategies to combat the pandemic and the infodemic.

In commonly used models for infectious diseases, infection is considered to be spreading in a population when *R*_0_ is greater than 1, and the epidemic is harder to control with a larger value of *R*_0_. Therefore, given the *R*_0_ of 7.62, it is reasonable to conclude that an infodemic is ongoing in our study population.

It is interesting that health beliefs involving perceived susceptibility increased in advance of the actual evolvement of the pandemic. Because we observed the basic reproduction number *R*_0_ of 7.62, suggestive of an infodemic, these findings may suggest that the volume of information regarding COVID-19 affects Twitter users’ perspectives regarding the risk of infection. In the early stages of the pandemic, before mortalities were observed, it is possible that less severity was assumed. Over time, perceived severity may have increased as the number of deaths cumulated. Strong correlations between perceived susceptibility and perceived severity regarding the case and death dynamics may suggest that the ongoing situation of the pandemic is a significant impact factor affecting health beliefs.

From the line chart in Figure 5, we observed a dramatic increase in daily cases between week 11 and week 14. There was also an upward trend in perceived susceptibility starting from week 11, which began decreasing by week 13. This phenomenon is interesting when we take the lockdown situation into consideration, as starting from week 13 was when most of the states were under the government-issued lockdown. Thus, official interventions were observed to potentially mitigate the general public’s perceived susceptibility of COVID-19. Meanwhile, we saw the growth of the number of confirmed cases slowing down during this same period. Interestingly, we failed to see a decline in perceived severity even when almost all the states were under quarantine. Previously, we showed that perceived severity was found to most strongly correlate with mortality; thus, it is reasonable that lockdown policies did not ease such health concerns, perhaps owing to the fact that while lockdowns slow down the spread of infection, they do not offer complete protection, especially in the absence of viable medications or treatment strategies.

As shown in Figure 6, for the first 3 to 4 weeks, topics predominately covered confirmed cases worldwide when the global pandemic was not yet affirmed by the authorities. In the following weeks, the terms *cruise ship* [29] and *li wenliang* [30] came into the spotlight. In early February, a large and notable cluster of COVID-19 cases occurred on the Diamond Princess cruise ship. Dr Wenliang Li, the Chinese doctor who tried to raise the alarm about a possible outbreak of a disease that resembled SARS-CoV-2 in Wuhan, China, died of the infection on February 7 , 2020. During weeks 8 and 9, when the COVID-19 outbreak heightened in Italy [31] and Iran [32], topics related to these countries began trending. On March 18, 2020 (ie, week 12), then President Trump announced that he was taking HCQ as prophylaxis for COVID-19 [33] and triggered massive discussions. In fact, starting at week 13, discussions involving HCQ and CQ began to dominate. Lastly, we were initially surprised to observe other topics like *george floyd* [34] in the health belief–related tweets. However, this topic is related to many events where people gathered that happened while many states were still under lockdown, possibly provoking health concerns. Through this analysis, we suspect that the news from all sources may penetrate into discussions regarding health beliefs and, thus, may influence health beliefs. Therefore, the news that we consume every day may inadvertently be a substantial factor that affects our health beliefs, which may contribute to and even exacerbate the infodemic on social media.

We observed that speeches by politicians could have dramatic impacts on the health beliefs of the general public who read the news. However, politicians’ speeches do not necessarily recapitulate scientific facts or evidence and could sometimes be misleading [35]. Thus, we expect to rely more on scientific sources, such as publications with scientific evidence or announcements made by health authorities, for more accurate and reliable information regarding the pandemic. However, it is uncertain whether scientific events or nonscientific events have a more profound influence on altering the health beliefs of the general public.

The results from the Kruskal-Wallis test imply that scientific events and nonscientific events did not significantly differ from one another in regard to their effect on health beliefs within the given period (ie, January 6 to June 21, 2020). We found it surprising that scientific events did not appear to be significantly associated with altering the health beliefs toward potential treatments in our data set. This might be due to the public’s distrust in science arising from the many uncertainties involving the pandemic or the instances of being delivered conflicting information, such as “Don't wear masks” to “Wear masks all the time.” To better cope with COVID-19 circumstances, everyone in society, online and offline, should be aware of the overabundance of information and its potential impact on health beliefs. Thus, it is essential to be prudent to screen the authenticity of each piece of information.

### Limitations and Future Work

We have identified some limitations in this study. The tweets analyzed in this study covered the English language, and there might be divergences across different languages that were not addressed. Although English tweets constitute the largest proportion among all the tweets, the number of tweets in other languages or undefined languages are still considerable [36]. We hope to expand the analysis to a multilingual setting in future work. Additionally, although we did not cover every potential treatment at this stage, our framework is extensible to assess the influence on health beliefs of additional treatments or interventions, such as vaccines. In fact, we plan to apply similar approaches to investigate health beliefs in COVID-19 vaccines once they are available to the general public. Furthermore, we likely have not considered other factors that may contribute to alterations in health beliefs.

This analysis used data extracted from one social media platform, Twitter, which may also introduce bias. Users’ health beliefs may not represent those of the entire population, since not everyone uses Twitter. More social media platforms will be incorporated in future work, such as Facebook, Instagram, and Reddit. Additionally, it would be interesting to compare our crowdsourcing results with health beliefs obtained through hospital-administrated surveys from patients with COVID-19 and their caretakers.

Technically, this study employed the very classic text classification methods, which used a combination of the bag-of-words model and machine learning classifiers. Yet, the experiments showed that they worked well (ie, AUROC curve over 0.9) on the given data. Deep learning architectures were not discussed in this study, mainly because there is no guarantee that deep learning models always work better than simple machine learning classifiers. Meanwhile, deep learning models bear higher technical barriers, which compromise the accessibility for people from other domains. Deep learning models are also known to demand considerable energy [37], so we were also trying to trade off the energy-performance balance. However, it is definitely worth a whole other study to discuss various NLP techniques for the classification task. For future studies, we are also interested in investigating the performance of various NLP techniques on the current text classification tasks.

### Conclusions

Our data suggest that we are not only fighting a pandemic but also an infodemic. The excessive information disseminated on social media platforms and other sources is closely related to the dynamics of the general public’s health beliefs. The dynamics of the pandemic, news, scientific and nonscientific events, and even the related tweets already published on social media platforms may influence the health beliefs of the general public on social media to some extent. Our findings provide clues and evidence for more effective management of the infodemic associated with the COVID-19 pandemic.

## Appendix

Multimedia Appendix 1 Supplementary information.

## Abbreviations

API: application programming interface
AUROC: area under the receiver operating characteristic
CQ: chloroquine
HBM: health belief model
HCQ: hydroxychloroquine
HPV: human papillomavirus
NLP: natural language processing
SIR: susceptible-infectious-recovered
tf-idf: term frequency–inverse document frequency
WHO: World Health Organization
