# Supplementary material for: Using Tweets to Understand How COVID-19–Related Health Beliefs Are Affected in the Age of Social Media: Twitter Data Analysis Study
Source: J Med Internet Res. 2021 Feb 22;23(2):e26302. doi: 10.2196/26302 (PMC7901597; doi:10.2196/26302)
Supplement: Multimedia Appendix 1 [file jmir_v23i2e26302_app1.docx]

# Supplementary Information

**Examples for spot checking of the classification**

In order to confirm the validity of the classifiers, we performed spot check for each HBM constructs. We randomly pulled samples for each health belief model (HBM) construct and showed one example for each construct here (all the tweets were converted to lowercase and all symbols were removed after pre-processing).

- Perceived susceptibility: “*singapore has recorded eight new cases of coronavirus, including one in prime minister lee hsien loong 's constituency, taking the total number of people infected by the deadly virus in the city state to 138*”
- Perceived severity: “*coronavirus hospital death toll rises to 148*”
- Perceived benefits: “*french microbiologist didier raoult released results of his large study of 1061 coronavirus patients with hydroxychloroquine raoult says the treatment had a strong outcome in 917 of patients he says 98 were eventually cured*”
- Perceived barriers: “*the fda issued a warning that malaria drugs touted by the president in treating covid 19 have been reported to have serious and life threatening side effects when used to treat the disease*”
- NOT HBM related: “*another reason for hospitals' reluctance to accept patients infected with the new coronavirus since treatment fees paid to hospitals under the medical insurance system are low for infectious diseases, accepting more of these patients will impact medical facilities' finances*”

# **Calculation of** $\boldsymbol{R}_{\boldsymbol{0}}$

We interpreted *S* as the number of users who tweeted anything about COVID-19, *I* as the number of users who tweeted about health beliefs, and *R* as the number of users who stopped tweeting about health beliefs; thus, *I + R* constitutes the number of users that have published a post regarding health beliefs. The SIR model is described as follows:

$$\begin{aligned} \partial_{t}S= -\beta S \cdot I/N\#\left( 1 \right) \end{aligned}$$

$$\begin{aligned} \partial_{t}I= \beta S \cdot I/N- \gamma I \#\left( 2 \right) \end{aligned}$$

$$\begin{aligned} \partial_{t}R= \gamma I \#\left( 3 \right) \end{aligned}$$

$$\begin{aligned} R_{0}= \beta/\gamma\#\left( 4 \right) \end{aligned}$$

In the original epidemiological model, the entire susceptible population of size N can be infected with a rate of $\beta$ by contacting infected individuals, and the infected individuals can recover with a rate $\gamma$.

**Performance of machine learning classifiers**

Classifiers: Ridge classifier, Perceptron, passive-aggressive classifier (Passive Aggressive), k-nearest neighbors classifier (kNN), Random Forest, Support Vector Machine with linear kernel and l1 (LinearSVC_L1) or l2 penalty (LinearSVC_L2), Support Vector Machine with rbf (SVC_rbf) or poly (SVC_poly) or sigmoid kernel (SVC_sigmoid), Stochastic Gradient Descent classifier with l1 (SGDClassifier_L1) or l2 (SGDClassifier_L2) or elastic net penalty (SGDClassifier_EN), multinomial naïve Bayesian classifier (MultinomialMB), Bernoulli naïve Bayesian classifier (BernoulliNB), and Logistic Regression.

# **Table S1. Performance for perceived susceptibility.**

| **Classifier** | **AUC^a^** | **Accuracy** | **Precision^b^** | **Recall^c^** | **F1^d^** |
| --- | --- | --- | --- | --- | --- |
| Ridge Classifier | 0.5000 | 0.8224 | 0.4112 | 0.5000 | 0.4513 |
| Perceptron | 0.9516 | 0.9173 | 0.8790 | 0.8235 | 0.8475 |
| Passive Aggressive | 0.9547 | 0.9234 | 0.8879 | 0.8379 | 0.8600 |
| kNN | 0.9413 | 0.9197 | 0.8818 | 0.8304 | 0.8529 |
| Random Forest | **0.9664** | 0.9367 | 0.9246 | 0.8549 | 0.8846 |
| LinearSVC_L1 | 0.9575 | 0.9367 | 0.8814 | 0.9132 | 0.8961 |
| LinearSVC_L2 | 0.9631 | 0.9258 | 0.8847 | 0.8529 | 0.8676 |
| SGDClassifier_L1 | 0.9507 | 0.9209 | 0.8897 | 0.8257 | 0.8529 |
| SGDClassifier_L2 | 0.9564 | 0.9258 | 0.8973 | 0.8367 | 0.8628 |
| SGDClassifier_EN | 0.9569 | 0.9234 | 0.8947 | 0.8299 | 0.8574 |
| MultinomialNB | 0.9310 | 0.8929 | 0.8974 | 0.7174 | 0.7673 |
| BernoulliNB | 0.9374 | 0.9185 | 0.8599 | 0.8618 | 0.8609 |
| Logistic Regression | 0.9655 | 0.9173 | 0.8456 | 0.9014 | 0.8693 |
| SVC_rbf | 0.9599 | 0.9331 | 0.9095 | 0.8519 | 0.8770 |
| SVC_poly | 0.9213 | 0.8978 | 0.8851 | 0.7419 | 0.7877 |
| SVC_sigmoid | 0.9651 | 0.9148 | 0.8400 | 0.9106 | 0.8685 |

# **Table S2. Performance for perceived severity.**

| **Classifier** | **AUC^a^** | **Accuracy** | **Precision^b^** | **Recall^c^** | **F1^d^** |
| --- | --- | --- | --- | --- | --- |
| Ridge Classifier | 0.5000 | 0.8224 | 0.4112 | 0.5000 | 0.4513 |
| Perceptron | 0.8961 | 0.8796 | 0.8429 | 0.7039 | 0.7451 |
| Passive Aggressive | **0.9176** | 0.9039 | 0.8785 | 0.7697 | 0.8093 |
| kNN | 0.8864 | 0.8577 | 0.9105 | 0.6020 | 0.6296 |
| Random Forest | 0.8901 | 0.8528 | 0.8785 | 0.7870 | 0.7658 |
| LinearSVC_L1 | 0.9077 | 0.8869 | 0.8506 | 0.7272 | 0.7675 |
| LinearSVC_L2 | 0.9137 | 0.8990 | 0.8697 | 0.7587 | 0.7982 |
| SGDClassifier_L1 | 0.8964 | 0.8856 | 0.8661 | 0.7103 | 0.7552 |
| SGDClassifier_L2 | 0.9018 | 0.8893 | 0.8671 | 0.7233 | 0.7674 |
| SGDClassifier_EN | 0.9011 | 0.8820 | 0.8405 | 0.7161 | 0.7557 |
| MultinomialNB | 0.8700 | 0.8650 | 0.8548 | 0.6413 | 0.6799 |
| BernoulliNB | 0.8694 | 0.8650 | 0.7688 | 0.7702 | 0.7695 |
| Logistic Regression | 0.8954 | 0.8491 | 0.7527 | 0.8331 | 0.7795 |
| SVC_rbf | 0.9014 | 0.9002 | 0.8742 | 0.7595 | 0.8000 |
| SVC_poly | 0.8679 | 0.8637 | 0.8153 | 0.6594 | 0.6967 |
| SVC_sigmoid | 0.9049 | 0.8504 | 0.7550 | 0.8392 | 0.7826 |

# **Table S3. Performance for perceived benefits.**

| **Classifier** | **AUC^a^** | **Accuracy** | **Precision^b^** | **Recall^c^** | **F1^d^** |
| --- | --- | --- | --- | --- | --- |
| Ridge Classifier | 0.8213 | 0.7652 | 0.7567 | 0.7500 | 0.7527 |
| Perceptron | 0.8082 | 0.7543 | 0.7474 | 0.7329 | 0.7374 |
| Passive Aggressive | 0.8224 | 0.7579 | 0.7500 | 0.7394 | 0.7432 |
| kNN | 0.7883 | 0.7238 | 0.7297 | 0.6835 | 0.6879 |
| Random Forest | **0.8675** | 0.7859 | 0.7775 | 0.7757 | 0.7766 |
| LinearSVC_L1 | 0.8223 | 0.7555 | 0.7474 | 0.7369 | 0.7406 |
| LinearSVC_L2 | 0.8215 | 0.7543 | 0.7448 | 0.7393 | 0.7416 |
| SGDClassifier_L1 | 0.8198 | 0.7628 | 0.7574 | 0.7410 | 0.7460 |
| SGDClassifier_L2 | 0.8221 | 0.7676 | 0.7599 | 0.7510 | 0.7544 |
| SGDClassifier_EN | 0.8230 | 0.7676 | 0.7599 | 0.7510 | 0.7544 |
| MultinomialNB | 0.7987 | 0.7409 | 0.7373 | 0.7122 | 0.7178 |
| BernoulliNB | 0.8121 | 0.7470 | 0.7384 | 0.7273 | 0.7310 |
| Logistic Regression | 0.8181 | 0.7506 | 0.7422 | 0.7483 | 0.7441 |
| SVC_rbf | 0.8430 | 0.7786 | 0.7705 | 0.7771 | 0.7727 |
| SVC_poly | 0.8220 | 0.7640 | 0.7585 | 0.7425 | 0.7475 |
| SVC_sigmoid | 0.8121 | 0.7470 | 0.7398 | 0.7472 | 0.7414 |

# **Table S4. Performance for perceived barriers.**

| **Classifier** | **AUC^a^** | **Accuracy** | **Precision^b^** | **Recall^c^** | **F1^d^** |
| --- | --- | --- | --- | --- | --- |
| Ridge Classifier | 0.8326 | 0.7628 | 0.7577 | 0.7544 | 0.7558 |
| Perceptron | 0.8233 | 0.7530 | 0.7481 | 0.7430 | 0.7449 |
| Passive Aggressive | 0.8327 | 0.7616 | 0.7578 | 0.7500 | 0.7526 |
| kNN | 0.8133 | 0.7360 | 0.7402 | 0.7135 | 0.7174 |
| Random Forest | **0.8612** | 0.7689 | 0.7663 | 0.7721 | 0.7668 |
| LinearSVC_L1 | 0.8344 | 0.7652 | 0.7606 | 0.7558 | 0.7577 |
| LinearSVC_L2 | 0.8403 | 0.7591 | 0.7541 | 0.7501 | 0.7517 |
| SGDClassifier_L1 | 0.8282 | 0.7652 | 0.7608 | 0.7554 | 0.7574 |
| SGDClassifier_L2 | 0.8284 | 0.7664 | 0.7621 | 0.7565 | 0.7586 |
| SGDClassifier_EN | 0.8287 | 0.7701 | 0.7655 | 0.7612 | 0.7629 |
| MultinomialNB | 0.8043 | 0.7275 | 0.7233 | 0.7121 | 0.7150 |
| BernoulliNB | 0.8253 | 0.7421 | 0.7364 | 0.7327 | 0.7342 |
| Logistic Regression | 0.8405 | 0.7603 | 0.7558 | 0.7598 | 0.7570 |
| SVC_rbf | 0.8475 | 0.7749 | 0.7701 | 0.7676 | 0.7687 |
| SVC_poly | 0.8321 | 0.7518 | 0.7475 | 0.7400 | 0.7426 |
| SVC_sigmoid | 0.8361 | 0.7579 | 0.7533 | 0.7573 | 0.7545 |

# **Table S5. Performance for HBM-related.**

| **Classifier** | **AUC^a^** | **Accuracy** | **Precision^b^** | **Recall^c^** | **F1^d^** |
| --- | --- | --- | --- | --- | --- |
| Ridge Classifier | 0.8982 | 0.8380 | 0.8401 | 0.8340 | 0.8358 |
| Perceptron | 0.8893 | 0.8260 | 0.8286 | 0.8215 | 0.8234 |
| Passive Aggressive | 0.8864 | 0.8510 | 0.8516 | 0.8483 | 0.8495 |
| kNN | 0.7134 | 0.7070 | 0.7159 | 0.7134 | 0.7068 |
| Random Forest | **0.9038** | 0.8410 | 0.8403 | 0.8395 | 0.8398 |
| LinearSVC_L1 | 0.9009 | 0.8280 | 0.8298 | 0.8240 | 0.8257 |
| LinearSVC_L2 | 0.8944 | 0.8420 | 0.8432 | 0.8387 | 0.8401 |
| SGDClassifier_L1 | 0.8940 | 0.8500 | 0.8524 | 0.8461 | 0.8480 |
| SGDClassifier_L2 | 0.8946 | 0.8500 | 0.8516 | 0.8466 | 0.8482 |
| SGDClassifier_EN | 0.8936 | 0.8500 | 0.8521 | 0.8463 | 0.8480 |
| MultinomialNB | 0.8194 | 0.7180 | 0.7197 | 0.7109 | 0.7119 |
| BernoulliNB | 0.8953 | 0.8230 | 0.8227 | 0.8247 | 0.8227 |
| Logistic Regression | 0.8955 | 0.8400 | 0.8396 | 0.8381 | 0.8387 |
| SVC_rbf | 0.8911 | 0.8480 | 0.8479 | 0.8458 | 0.8466 |
| SVC_poly | 0.8603 | 0.7920 | 0.7912 | 0.7928 | 0.7914 |
| SVC_sigmoid | 0.8751 | 0.8310 | 0.8299 | 0.8311 | 0.8303 |

^a^ AUC: Area Under the Receiver Operating Characteristic Curve (the best AUCs are highlighted in bold)
^b^ Macro averaged precision
^c^ Macro averaged recall
^d^ Macro averaged F1 score

# **Table S6. Official documents for lockdown and reopen in the US.**

| **State** | **Governor** | **Lockdown  date** | **Reference** | **Reopen  date** | **Reference** | **Note** |
| --- | --- | --- | --- | --- | --- | --- |
| Puerto Rico | Wanda Vázquez-Garced | 15-Mar | <https://basecero.ogp.pr.gov/apex/apex_util.get_blob?s=16003349125019&a=161&c=112063554695324788&p=15&k1=4725&k2=&ck=kD2_DzwNpUQzd5je4I86X_5-auqRn24Tgi5KAcF0dwirxCFn7Cjd7TpUHUF5OPzc2tGJ7NGQbw7WVVuvbHO8pw&rt=IR> | 26-May | <https://basecero.ogp.pr.gov/apex/apex_util.get_blob?s=16003349125019&a=161&c=112063554695324788&p=15&k1=4776&k2=&ck=ii-DWkxob-RPm4U_iLaUdA30HQEemW3SOUyb_ynJt-JM5329guelpQyZkt4iQR0wNmBrRmKX1BJUqKYVTF5E8Q&rt=IR> |  |
| California | Gavin Newsom | 19-Mar | <https://www.gov.ca.gov/wp-content/uploads/2020/03/3.19.20-attested-EO-N-33-20-COVID-19-HEALTH-ORDER.pdf> | 25-May | [https://www.cdph.ca.gov/Programs/OPA/Pages/NR20-100.aspx#](https://www.cdph.ca.gov/Programs/OPA/Pages/NR20-100.aspx) |  |
| Illinois | JB Pritzker | 21-Mar | <https://www2.illinois.gov/Documents/ExecOrders/2020/ExecutiveOrder-2020-10.pdf> | 29-May | <https://www2.illinois.gov/Documents/ExecOrders/2020/ExecutiveOrder-2020-38.pdf> |  |
| New Jersey | Philip D. Murphy | 21-Mar | <https://nj.gov/infobank/eo/056murphy/pdf/EO-107.pdf> | 9-Jun | <http://d31hzlhk6di2h5.cloudfront.net/20200609/7b/4b/9c/2f/be885a1520042ccdf9320476/EO-152.pdf> |  |
| New York | Andrew M. Cuomo | 22-Mar | <https://on.ny.gov/2U4UVKh> | 15-May | <https://on.ny.gov/3ck80Gc> |  |
| Washington | Jay Inslee | 23-Mar | [https://www.governor.wa.gov/sites/default/files/proclamations/20-25 Coronovirus Stay Safe-Stay Healthy (tmp) (002).pdf](https://www.governor.wa.gov/sites/default/files/proclamations/20-25%20Coronovirus%20Stay%20Safe-Stay%20Healthy%20(tmp)%20(002).pdf) | 4-May | [https://www.governor.wa.gov/sites/default/files/20-25.3 - COVID-19 Stay Home Stay Healthy - Reopening (tmp).pdf](https://www.governor.wa.gov/sites/default/files/20-25.3%20-%20COVID-19%20Stay%20Home%20Stay%20Healthy%20-%20Reopening%20(tmp).pdf) |  |
| Louisiana | John Bel Edwards | 23-Mar | <https://gov.louisiana.gov/assets/Proclamations/2020/JBE-33-2020.pdf> | 15-May | <https://gov.louisiana.gov/assets/Proclamations/2020/59-JBE-2020.pdf> |  |
| Oregon | Kate Brown | 23-Mar | [https://govsite-assets.s3.amazonaws.com/jkAULYKcSh6DoDF8wBM0_EO 20-12.pdf](https://govsite-assets.s3.amazonaws.com/jkAULYKcSh6DoDF8wBM0_EO%2020-12.pdf) | 15-May | <https://www.oregon.gov/gov/Documents/executive_orders/eo_20-25.pdf> |  |
| Ohio | Mike DeWine | 23-Mar | [https://content.govdelivery.com/attachments/OHOOD/2020/03/22/file_attachments/1407840/Stay Home Order.pdf](https://content.govdelivery.com/attachments/OHOOD/2020/03/22/file_attachments/1407840/Stay%20Home%20Order.pdf) | 15-May | <https://coronavirus.ohio.gov/static/publicorders/Directors-Order-Personal-Services.pdf> |  |
| Connecticut | Ned Lamont | 23-Mar | <https://portal.ct.gov/-/media/Office-of-the-Governor/Executive-Orders/Lamont-Executive-Orders/Executive-Order-No-7H.pdf> | 20-May | <https://portal.ct.gov/-/media/DECD/Covid_Business_Recovery/CTReopens_Restaurants_C5_V1.pdf?la=en> |  |
| Indiana | Eric J. Holcomb | 24-Mar | <https://www.in.gov/gov/files/Executive_Order_20-08_Stay_at_Home.pdf> | 4-May | <https://www.backontrack.in.gov/2362.htm> |  |
| West Virginia | Jim Justice | 24-Mar | <https://coronavirus-wvgovstatus-cdn.azureedge.net/STAY_AT_HOME_ORDER.pdf> | 4-May | [https://governor.wv.gov/Documents/2020 Executive Orders/Executive-Order-April-30-2020-Safer-At-Home-Order.pdf](https://governor.wv.gov/Documents/2020%20Executive%20Orders/Executive-Order-April-30-2020-Safer-At-Home-Order.pdf) |  |
| New Mexico | Michelle Lujan Grisham | 24-Mar | <https://www.newmexico.gov/2020/03/23/state-enacts-further-restrictions-to-stop-spread-including-stay-at-home-instruction/> | 16-May | <https://www.governor.state.nm.us/2020/05/13/state-to-further-modify-public-health-emergency-order/> |  |
| Massachusetts | Charlie Baker | 24-Mar | <https://www.mass.gov/doc/march-23-2020-essential-services-and-revised-gatherings-order/download> | 18-May | <https://www.mass.gov/doc/signed-second-extension-of-essential-services-order/download> |  |
| Michigan | Gretchen Whitmer | 24-Mar | <https://www.michigan.gov/whitmer/0,9309,7-387-90499_90705-522626--,00.html> | 1-Jun | <https://www.michigan.gov/whitmer/0,9309,7-387-90499_90705-530620--,00.html> |  |
| Delaware | John C. Carney | 24-Mar | <https://governor.delaware.gov/wp-content/uploads/sites/24/2020/03/Fifth-Modification-to-State-of-Emergency-03222020.pdf> | 1-Jun | <https://governor.delaware.gov/wp-content/uploads/sites/24/2020/09/Twentieth-Modification-to-State-of-Emergency_05312020.pdf> |  |
| Oklahoma | Kevin Stitt | 25-Mar | <https://www.sos.ok.gov/documents/executive/1926.pdf> | 24-Apr | <https://www.sos.ok.gov/documents/executive/1937.pdf> |  |
| Idaho | Brad Little | 25-Mar | <https://coronavirus.idaho.gov/wp-content/uploads/2020/06/statewide-stay-home-order_032520.pdf> | 1-May | <https://coronavirus.idaho.gov/wp-content/uploads/2020/06/amended-statewide-stay-home-order_041520.pdf> |  |
| Wyoming | Mark Gordon | 25-Mar | <https://governor.wyo.gov/media/news-releases/2020-news-releases/gov-gordon-top-officials-issue-plea-for-wyoming-citizens-to-stay-home-whe> | 1-May | <https://governor.wyo.gov/media/news-releases/2020-news-releases/governor-gordon-authorizes-re-opening-of-gyms-personal-care-services-under> | plea |
| Hawaii | David Ige | 25-Mar | <https://governor.hawaii.gov/wp-content/uploads/2020/03/2003162-ATG_Third-Supplementary-Proclamation-for-COVID-19-signed.pdf> | 7-May | <https://governor.hawaii.gov/wp-content/uploads/2020/05/2005024-ATG_Seventh-Supplementary-Proclamation-for-COVID-19-distribution-signed-1.pdf> |  |
| Wisconsin |  | 25-Mar | <https://evers.wi.gov/Documents/COVID19/EMO12-SaferAtHome.pdf> | 13-May | <https://content.govdelivery.com/accounts/WIGOV/bulletins/28b7302> |  |
| Vermont | Philip B. Scott | 25-Mar | [https://governor.vermont.gov/sites/scott/files/documents/ADDENDUM 6 TO EXECUTIVE ORDER 01-20.pdf](https://governor.vermont.gov/sites/scott/files/documents/ADDENDUM%206%20TO%20EXECUTIVE%20ORDER%2001-20.pdf) | 15-May | [https://governor.vermont.gov/sites/scott/files/documents/ADDENDUM 14 TO EXECUTIVE ORDER 01-20.pdf](https://governor.vermont.gov/sites/scott/files/documents/ADDENDUM%2014%20TO%20EXECUTIVE%20ORDER%2001-20.pdf) |  |
| Colorado | Jared Polis | 26-Mar | [https://www.colorado.gov/governor/sites/default/files/inline-files/D 2020 017 Ordering Coloradans to Stay at Home_0.pdf](https://www.colorado.gov/governor/sites/default/files/inline-files/D%202020%20017%20Ordering%20Coloradans%20to%20Stay%20at%20Home_0.pdf) | 27-Apr | https://www.colorado.gov/governor/sites/default/files/inline-files/D 2020 044 Safer at Home.pdf |  |
| Kentucky | Andy Beshear | 26-Mar | <https://governor.ky.gov/attachments/20200325_Executive-Order_2020-257_Healthy-at-Home.pdf> | 11-May | <https://governor.ky.gov/attachments/20200508_Executive-Order_2020-323_Reopening.pdf> |  |
| Utah | Gary R. Herbert | 27-Mar | [https://coronavirus-download.utah.gov/Governor/Stay At Home Declaration 1.pdf](https://coronavirus-download.utah.gov/Governor/Stay%20At%20Home%20Declaration%201.pdf) | 1-May | https://coronavirus-download.utah.gov/Governor/Stay Safe Stay Home.20.04.17.pdf | directive |
| New Hampshire | Chris Sununu | 27-Mar | <https://www.state.gov/wp-content/uploads/2020/03/2020-03-29-Notice-New-Hampshire-Stay-Home-Order.pdf> | 11-May | <https://www.governor.nh.gov/news-and-media/governor-chris-sununu-announces-stay-home-20> |  |
| Minnesota | Tim Walz | 27-Mar | [https://mn.gov/governor/assets/3a. EO 20-20 FINAL SIGNED Filed_tcm1055-425020.pdf](https://mn.gov/governor/assets/3a.%20EO%2020-20%20FINAL%20SIGNED%20Filed_tcm1055-425020.pdf) | 18-May | [https://mn.gov/governor/assets/EO 20-56 Final_tcm1055-433768.pdf?TSPD_101_R0=a0a69313324b5de1221521392f4c1a8abe0000000000000000015a27ca5ffff00000000000000000000000000005f9e8d0d002179dd92](https://mn.gov/governor/assets/EO%2020-56%20Final_tcm1055-433768.pdf?TSPD_101_R0=a0a69313324b5de1221521392f4c1a8abe0000000000000000015a27ca5ffff00000000000000000000000000005f9e8d0d002179dd92) |  |
| Alaska | Michael J. Dunleavy | 28-Mar | <https://gov.alaska.gov/wp-content/uploads/sites/2/03272020-SOA-COVID-19-Health-Mandate-012.pdf> | 24-Apr | <https://gov.alaska.gov/newsroom/2020/04/21/governor-unveils-path-to-reopening-alaskan-economy/> |  |
| Montana | Steve Bullock | 28-Mar | [https://covid19.mt.gov/Portals/223/Documents/Stay at Home Directive.pdf?ver=2020-03-26-173332-177](https://covid19.mt.gov/Portals/223/Documents/Stay%20at%20Home%20Directive.pdf?ver=2020-03-26-173332-177) | 26-Apr | <https://dphhs.mt.gov/aboutus/news/2020/phasedreopening> |  |
| Rhode Island | Gina M. Raimondo | 28-Mar | <https://governor.ri.gov/documents/orders/Executive-Order-20-14.pdf> | 9-May | <https://governor.ri.gov/documents/orders/Executive-Order-20-32.pdf> |  |
| Kansas | Laura Kelly | 30-Mar | <https://governor.kansas.gov/wp-content/uploads/2020/03/EO20-16.pdf> | 4-May | <https://governor.kansas.gov/wp-content/uploads/2020/04/EO-20-29-Implementing-Phase-One-of-Ad-Astra-Plan.pdf> |  |
| North Carolina | Roy Cooper | 30-Mar | <https://files.nc.gov/governor/documents/files/EO121-Stay-at-Home-Order-3.pdf> | 8-May | <https://files.nc.gov/governor/documents/files/EO135-Extensions.pdf> |  |
| Maryland | Larry Hogan | 30-Mar | <https://governor.maryland.gov/wp-content/uploads/2020/03/Gatherings-FOURTH-AMENDED-3.30.20.pdf> | 15-May | <https://governor.maryland.gov/wp-content/uploads/2020/05/Gatherings-SIXTH-AMENDED-5.13.20.pdf> |  |
| Virginia | Ralph S. Northam | 30-Mar | <https://www.governor.virginia.gov/media/governorvirginiagov/executive-actions/EO-55-Temporary-Stay-at-Home-Order-Due-to-Novel-Coronavirus-(COVID-19).pdf> | 15-May | <https://www.governor.virginia.gov/media/governorvirginiagov/executive-actions/EO-62-and-Order-of-Public-Health-Emergency-Four-AMENDED.pdf> |  |
| Tennessee | Bill Lee | 31-Mar | <https://publications.tnsosfiles.com/pub/execorders/exec-orders-lee22.pdf> | 27-Apr | <https://publications.tnsosfiles.com/pub/execorders/exec-orders-lee29.pdf> |  |
| Arizona | Douglas A. Ducey | 31-Mar | <https://www.azdhs.gov/documents/preparedness/epidemiology-disease-control/infectious-disease-epidemiology/novel-coronavirus/eo-stay-home-stay-healthy-stay-connected.pdf> | 8-May | <https://azgovernor.gov/sites/default/files/eo_2020-33_0.pdf> |  |
| Pennsylvania | Tom Wolf | 1-Apr | <https://www.governor.pa.gov/wp-content/uploads/2020/04/03.23.20-Stay-At-Home-Order-Guidance.pdf> | 8-May | <https://www.governor.pa.gov/newsroom/gov-wolf-announces-reopening-of-24-counties-beginning-may-8/> |  |
| Nevada | Steve Sisolak | 1-Apr | <https://gov.nv.gov/uploadedFiles/govnewnvgov/Content/News/Emergency_Orders/2020_attachments/010-Gov-Announces-Stay-at-Home-directive-extends-closure-date-to-the-end-of-April.pdf> | 9-May | <https://nvhealthresponse.nv.gov/wp-content/uploads/2020/05/Press-release-Phase-1-Reopening-Details.pdf> |  |
| District of Columbia | Muriel Bowser | 1-Apr | <https://coronavirus.dc.gov/release/mayor-bowser-issues-stay-home-order> | 29-May | <https://coronavirus.dc.gov/phaseone> |  |
| Texas | Greg Abbott | 2-Apr | <https://gov.texas.gov/uploads/files/press/EO-GA-14_Statewide_Essential_Service_and_Activity_COVID-19_IMAGE_03-31-2020.pdf> | 1-May | <https://gov.texas.gov/uploads/files/press/EO-GA-18_expanded_reopening_of_services_COVID-19.pdf> |  |
| Maine | Janet T. Mills | 2-Apr | [https://www.maine.gov/governor/mills/sites/maine.gov.governor.mills/files/inline-files/CORRECTED_An Order Regarding Further Restrictions on Public Contact and Movement, Schools, Vehicle Travel and Retail Business Operations.pdf](https://www.maine.gov/governor/mills/sites/maine.gov.governor.mills/files/inline-files/CORRECTED_An%20Order%20Regarding%20Further%20Restrictions%20on%20Public%20Contact%20and%20Movement,%20Schools,%20Vehicle%20Travel%20and%20Retail%20Business%20Operations.pdf) | 1-May | [https://www.maine.gov/governor/mills/sites/maine.gov.governor.mills/files/inline-files/An Order to Stay Safer at Home.pdf](https://www.maine.gov/governor/mills/sites/maine.gov.governor.mills/files/inline-files/An%20Order%20to%20Stay%20Safer%20at%20Home.pdf) |  |
| Georgia | Brain p. Kemp | 3-Apr | <https://gov.georgia.gov/document/2020-executive-order/04022001/download> | 24-Apr | <https://gov.georgia.gov/document/2020-executive-order/04202001/download> |  |
| Mississippi | Tate Reeves | 3-Apr | <https://www.sos.ms.gov/content/executiveorders/ExecutiveOrders/1466.pdf> | 27-Apr | <https://www.sos.ms.gov/content/executiveorders/ExecutiveOrders/1477.pdf> |  |
| Florida | Ron Desantis | 3-Apr | <https://www.flgov.com/wp-content/uploads/orders/2020/EO_20-91-compressed.pdf> | 4-May | <https://www.flgov.com/wp-content/uploads/orders/2020/EO_20-112.pdf> |  |
| Alabama | Kay Ivey | 4-Apr | <https://governor.alabama.gov/assets/2020/04/Final-Statewide-Order-4.3.2020.pdf> | 30-Apr | <https://governor.alabama.gov/assets/2020/04/Safer-At-Home-Order-Signed-4.28.20.pdf> |  |
| Missouri | Michael L. Parson | 6-Apr | <https://governor.mo.gov/priorities/stay-home-order> | 4-May | [https://content.govdelivery.com/attachments/MOGOV/2020/04/27/file_attachments/1437097/Economic Reopening Order 4-27-20.pdf](https://content.govdelivery.com/attachments/MOGOV/2020/04/27/file_attachments/1437097/Economic%20Reopening%20Order%204-27-20.pdf) |  |
| South Carolina | Henry Mcmaster | 7-Apr | [https://governor.sc.gov/sites/default/files/Documents/Executive-Orders/2020-04-06 eFILED Executive Order No. 2020-21 - Stay at Home or Work Order.pdf](https://governor.sc.gov/sites/default/files/Documents/Executive-Orders/2020-04-06%20eFILED%20Executive%20Order%20No.%202020-21%20-%20Stay%20at%20Home%20or%20Work%20Order.pdf) | 20-Apr | [https://governor.sc.gov/sites/default/files/Documents/Executive-Orders/2020-04-20 FILED Executive Order No. 2020-28 - Modification of Restrictions for Public Beaches & Waters & Incremental Modification of Non-Essential Business Closures.pdf](https://governor.sc.gov/sites/default/files/Documents/Executive-Orders/2020-04-20%20FILED%20Executive%20Order%20No.%202020-28%20-%20Modification%20of%20Restrictions%20for%20Public%20Beaches%20&%20Waters%20&%20Incremental%20Modification%20of%20Non-Essential%20Business%20Closures.pdf) |  |

*All the online documents were accessed on Oct 31, 2020.

# **Table S7. References for scientific and non-scientific events**

| June 15: FDA revokes its emergency use authorization of HCQ ^1^. |
| --- |
| June 5: Lancet paper published on May 22 is retracted ^2^. |
| June 3: WHO resumes study of HCQ for treating COVID-19 ^3^. |
| June 2: Richard Horton, editor of The Lancet, tweets about the validity of the study the medical journal published May 22 ^4^. |
| May 24: Trump says he's finished taking HCQ and is *"still here" ^5^.* |
| May 22: Lancet paper *"Hydroxychloroquine or chloroquine with or without a macrolide for treatment of COVID-19: a multinational registry analysis"* is published ^6^. |
| May 18: Trump says he is been taking HCQ ^7^. |
| May 14: NIH begins clinical trial of HCQ to treat COVID-19 ^8^. |
| May 11: JAMA study shows HCQ associated with cardiac arrest ^9^. |
| May 7: NEJM paper *"Observational Study of Hydroxychloroquine in Hospitalized Patients with Covid-19"* is published which shows no lowered risks taking HCQ ^10^. |
| May 5: News on Dr. Bright, former director of BARDA is fired after he resisted a push from HHS officials for widespread HCQ ^11^. |
| April 26: News on despite the FDA warning, many states stockpile HCQ ^12^. |
| April 24: FDA warns against HCQ use outside hospitals ^13^. |
| April 21: VA study shows no benefit of HCQ for COVID-19 ^14^. |
| April 20: Novartis starts large clinical trial of HCQ in hospitalized covid-19 patients ^15^. |
| April 14: Trump touts HCQ in meeting with recovered patients ^16^. |
| April 13: Study in Brazil links HCQ to fatal heart problems makes headlines ^17^. |
| April 9: NIH begins clinical trials of HCQ ^18^. |
| April 8: Medical societies warn of the use of HCQ for COVID-19 ^19^. |
| April 7: India lifts ban on HCQ exports ^20^. |
| April 5: Trump touts HCQ: *"What really do we have to lose?"* ^21^. |
| April 3: French study is retracted as publisher claims data *"did not meet its standards"* ^22^. |
| March 28: FDA approves emergency use of HCQ ^23^. |
| March 24: News on Arizona man dies after ingesting non-medication chloroquine ^24^. |
| March 21: Trump cites the success of a small French study ^25^ published on March 17 and touts HCQ ^26^. |
| March 20: Trump refutes as Dr. Fauci calls evidence on HCQ "anecdotal' on a task force briefing ^27^. |
| March 19: Trump declares HCQ a "game changer" ^28^. |
| March 17: Study *"Hydroxychloroquine and azithromycin as a treatment of COVID-19: results of an openlabel non-randomized clinical trial"* is published ^25^. |

References for Table S7.

1 RADM Denise M. Hinton. *FDA Revokes Emergency Use Authorization for Chloroquine and Hydroxychloroquine*, <<https://www.fda.gov/media/138945/download>> (2020).

2 Mehra, M. R., Desai, S. S., Ruschitzka, F. & Patel, A. N. RETRACTED: Hydroxychloroquine or chloroquine with or without a macrolide for treatment of COVID-19: a multinational registry analysis. *The Lancet*, doi:10.1016/S0140-6736(20)31180-6.

3 Alice Park. *WHO Resumes Study of Hydroxychloroquine for Treating COVID-19*, <<https://time.com/5847664/who-hydroxychloroquine-covid-19/>> (2020).

4 Richard Horton. in *Here is The Lancet’s Expression of Concern regarding the paper by Mandeep Mehra et al. Serious questions have been raised about the reliability of the findings reported in this paper.* [*https://twitter.com/TheLancet/status/1267901995848957953?s=20*](https://twitter.com/TheLancet/status/1267901995848957953?s=20) (Twitter, 2020).

5 Full Measure with Sharyl Attkisson. in *This week, an exclusive extended interview with President Trump. Recorded at the White House on Friday, May 22nd.* (Youtube, 2020).

6 Mehra, M. R., Desai, S. S., Ruschitzka, F. & Patel, A. N. Hydroxychloroquine or chloroquine with or without a macrolide for treatment of COVID-19: a multinational registry analysis. *The Lancet* (2020).

7 Berkeley Lovelas Jr. & Kevin Breuninger. *Trump says he takes hydroxychloroquine to prevent coronavirus infection even though it’s an unproven treatment*, <<https://www.cnbc.com/2020/05/18/trump-says-he-takes-hydroxychloroquine-to-prevent-coronavirus-infection.html>> (2020).

8 National Institutes of Health. *NIH begins clinical trial of hydroxychloroquine and azithromycin to treat COVID-19*, <<https://www.nih.gov/news-events/news-releases/nih-begins-clinical-trial-hydroxychloroquine-azithromycin-treat-covid-19>> (2020).

9 Rosenberg, E. S. *et al.* Association of treatment with hydroxychloroquine or azithromycin with in-hospital mortality in patients with COVID-19 in New York state. *Jama* (2020).

10 Geleris, J. *et al.* Observational study of hydroxychloroquine in hospitalized patients with Covid-19. *New England Journal of Medicine* (2020).

11 Maia Anderson. *Former fed official files whistleblower complaint against HHS*, <<https://www.beckershospitalreview.com/pharmacy/former-fed-official-files-whistleblower-complaint-against-hhs.html>> (2020).

12 Brandy McCombs & Lindsay Whitehurst. *U.S. states build stockpiles of malaria drug touted by Trump*, <<https://apnews.com/article/f249bde40d0c676ba5330e4373ba207c>> (2020).

13 FDA Drug Safety Podcast. *FDA cautions against use of hydroxychloroquine or chloroquine for COVID-19 outside of the hospital setting or a clinical trial due to risk of heart rhythm problems*, <<https://www.fda.gov/drugs/fda-drug-safety-podcasts/fda-cautions-against-use-hydroxychloroquine-or-chloroquine-covid-19-outside-hospital-setting-or>> (2020).

14 Magagnoli, J. *et al.* Outcomes of hydroxychloroquine usage in United States veterans hospitalized with Covid-19. *medRxiv*, 2020.2004.2016.20065920, doi:10.1101/2020.04.16.20065920 (2020).

15 Novatis. *Novartis to sponsor large clinical trial of hydroxychloroquine in hospitalized COVID-19 patients*, <<https://www.novartis.com/news/media-releases/novartis-sponsor-large-clinical-trial-hydroxychloroquine-hospitalized-covid-19-patients#:~:text=Basel%2C%20April%2020%2C%202020%20%E2%80%94,patients%20with%20COVID%2D19%20disease>.> (2020).

16 The White House. *Remarks by President Trump in a Meeting with Recovered COVID-19 Patients*, <<https://www.whitehouse.gov/briefings-statements/remarks-president-trump-meeting-recovered-covid-19-patients/>> (2020).

17 Borba, M. G. S. *et al.* Effect of high vs low doses of chloroquine diphosphate as adjunctive therapy for patients hospitalized with severe acute respiratory syndrome coronavirus 2 (SARS-CoV-2) infection: a randomized clinical trial. *JAMA network open* **3**, e208857-e208857 (2020).

18 National Institutes of Health. *NIH clinical trial of hydroxychloroquine, a potential therapy for COVID-19, begins*, <<https://www.nih.gov/news-events/news-releases/nih-clinical-trial-hydroxychloroquine-potential-therapy-covid-19-begins>> (2020).

19 American Heart Association. *Caution recommended on COVID-19 treatment with hydroxychloroquine and azithromycin for patients with cardiovascular disease*, <<https://newsroom.heart.org/news/caution-recommended-on-covid-19-treatment-with-hydroxychloroquine-and-azithromycin-for-patients-with-cardiovascular-disease-6797342>> (2020).

20 Archana Chaudhary. *India Partially Lifts Export Ban on Potential Coronavirus Treatment After Trump Call*, <<https://time.com/5816617/india-lifts-hydroxychloroquine-export-ban/>> (2020).

21 The White House. *Remarks by President Trump, Vice President Pence, and Members of the Coronavirus Task Force in Press Briefing*, <<https://www.whitehouse.gov/briefings-statements/remarks-president-trump-vice-president-pence-members-coronavirus-task-force-press-briefing-20/>> (2020).

22 Andreas Voss. *Official Statement from International Society of Antimicrobial Chemotherapy (ISAC)*, <<https://www.isac.world/news-and-publications/official-isac-statement>> (2020).

23 RADM Denise M. Hinton. (ed Food and Drug Administration) (2020).

24 Anne Flaherty & Sophie Tatum. *Man dies after ingesting aquarium product containing chloroquine: Hospital network*, <<https://abcnews.go.com/Politics/man-dies-ingesting-chloroquine-prevent-coronavirus-banner-health/story?id=69759570>> (2020).

25 Gautret, P. *et al.* Hydroxychloroquine and azithromycin as a treatment of COVID-19: results of an open-label non-randomized clinical trial. *International journal of antimicrobial agents*, 105949 (2020).

26 Donald J. Trump. in *HYDROXYCHLOROQUINE & AZITHROMYCIN, taken together, have a real chance to be one of the biggest game changers in the history of medicine. The FDA has moved mountains - Thank You! Hopefully they will BOTH (H works better with A, International Journal of Antimicrobial Agents).....* (Twitter, 2020).

27 The White House. *Remarks by President Trump, Vice President Pence, and Members of the C oronavirus Task Force in Press Briefing*, <<https://www.whitehouse.gov/briefings-statements/remarks-president-trump-vice-president-pence-members-c-oronavirus-task-force-press-briefing/>> (2020).

28 Stephanie Ebbs. *Trump announces potential 'game changer' on drugs to treat novel coronavirus, but FDA says more study is needed*, <<https://abcnews.go.com/Politics/trump-announces-potential-game-changer-drugs-treat-covid19/story?id=69693560>> (2020).
